# Extreme performance of multi-layer laminated glass designs under blast loads

**DOI:** 10.1038/s41598-025-04140-y

**Published:** 2025-07-01

**Authors:** Andrew Bowman, Alaa El-Sisi, Ahmed Elbelbisi, Ahmed Elkilani, Hesham Elemam, Hani Salim

**Affiliations:** 1 U.S. Army Engineer Research and Development Center, Vicksburg, MS 39180 USA; 2 Civil Engineering, Southern Illinois University Edwardsville, Edwardsville, IL 62025 USA; 3 Civil and Environmental Engineering, University of Missouri, Columbia, MO 65211 USA; 4 Civil Engineering Dapartment, Zagazig University, Zagazig, 44519 Egypt; 5 Structural Engineering, Hutchison Engineering Inc., Hannibal, Missouri, 63401 USA

**Keywords:** Laminated glass, Hybrid interlayer, Multi-layer types, Blast loads, Finite element modeling, Civil engineering, Glasses

## Abstract

Laminated Glass (LG) is a safety glass made by bonding multiple glass layers together with a polymeric interlayer, offering protection against flying shards, especially in explosive scenarios. In this paper, a numerical study was performed to study the effect of the laminated glass cross-section on the blast performance of the panel. Different configurations were studied, such as a double glass layer with interlayers, a double glass layer with hybrid interlayers, and a variety of multi-glass layer configurations with interlayers. Variations of glass layer thicknesses, number of glass layers, and order of glass layers were studied to evaluate the optimal design of the LG panel. Four different polymer interlayers were considered in this analysis: polyvinyl butyral (PVB), ethylene vinyl acetate (EVA), thermoplastic polyurethane (TPU), and the ionomer SentryGlas® (SG). A high-fidelity numerical model, capable of capturing damage and fracture of the LG, was employed using the Arbitrary Lagrangian–Eulerian three-dimensional analysis (ALE3D) multiphysics software, and blast load simulations were developed and verified against experimental data from the literature. Results show that interlayer type affects blast resistance differently, with EVA panels exhibiting the highest deformation and SG panels the least, indicating higher strength and better performance in SG interlayer panels. Increasing interlayer thickness enhances LG panel resistance in the pre-cracked stage. It was found that SG performs better than PVB and much better than TPU/EVA. EVA and TPU performed poorly and completely tore under the used blast loading. Hybrid interlayers underperformed, possibly due to unbalanced load sharing between the stiffer middle interlayer and the more compliant EVA outer membrane. Finally, unique combinations of glass thicknesses and configurations were studied and indicate that LG systems with a thick middle layer and thin outer layers deflect the least during a blast load. These findings underscore the critical role of layup design in enhancing the blast resistance of multi-layer LG panels.

## Introduction

In recent years, the importance of protection from extreme loading environments, such as impact and blast loading, has been recognized in structural design^1–6^. Window systems, which predominantly consist of annealed float glass, are particularly vulnerable in structural facades. Glass, being a brittle material, undergoes sudden fragmentation when it fails, resulting in numerous sharp fragments. To enhance the safety of window systems against explosions or impacts, laminated glass (LG) has emerged as an alternative. LG comprises two or more glass layers that are bonded using a polymer interlayer, such as polyvinyl butyral (PVB), ethylene vinyl acetate (EVA), thermoplastic polyurethane (TPU), or ionomers like SentryGlas® (SG)^7,8^. Once all the glass panes have cracked, the structural performance of the LG system is primarily governed by the resistance offered by the polymeric interlayer. This interlayer significantly enhances the system’s ductility and contributes substantially to its overall resistance during dynamic events. Consequently, understanding the static and dynamic properties of the interlayer is crucial for predicting the system’s performance under blast conditions^9^.

The polymer interlayer serves to retain the fragments of broken glass and increase the window system loading resistance. When a flexible polymer like PVB is utilized, the interlayer deforms and absorbs energy, thereby reducing the transmission of energy to the rest of the structure^10^.

Extensive research has been conducted on the post-fracture behavior of LG under the effect of blast loading^11^. The available literature encompasses numerous experimental studies on LG loaded by the blast, including shock tube tests^12–14^ and field tests^15–18^. The combined effect of blast loading and fragment impact on LG was investigated in a recent experimental study^11^. Finite element (FE) modeling was employed by several researchers to replicate the observed mechanical behavior in experiments^14–16,18^. A field study^19^ investigated the blast resistance of full-scale LG panels. The panels were tested with equivalent TNT charges of 15 and 30 kg at varying distances ranging from 10,000 mm to 16,000 mm. To comprehensively analyze their behavior, FE simulations using Abaqus captured the pre-crack and post-crack responses of the panels.

Larcher et al. compared different modeling approaches, including shell element, solid element, and smeared models, which showed that the most accurate results were evaluated using the solid element^10^. Hooper et al. developed a shell element model that assigned zero stiffness to the glass layer upon fracture, yielding results comparable to experimental data^16^. Zhang et al. investigated the failure mechanisms of laminated window systems through FE simulations and emphasized the dominant influence of boundary conditions on the behavior^18^. A comprehensive FE model for blast-loaded LG was developed to explore the possibility of delamination between the interlayer and glass^14^. The numerical model effectively captured various post-fracture behavior aspects. It was highlighted that it is necessary to generate a fine mesh to accurately model delamination and fracture processes if element erosion is employed^14^.

Extensive FE models have also been developed to assess the response of LG windows and glazing structures subjected to blast events. These models aim to improve blast resistance and optimize structural responses^20,21^ In an important study, the pre-cracked LG specimens subjected to tensile loading were studied with experimental and numerical investigation^22^. It was observed that the delamination energy, which represents an indication of the delamination process, depends on the loading rate^22^. Similar experiments conducted by Franz and Schneider demonstrated the strong correlation between delamination properties and the adhesion level between the PVB interlayer and the glass^23^. The properties of delamination were also found to be influenced by temperature^24^. Additionally, it is important to consider the probabilistic fracture strength of glass, which arises from microscopic flaws present on the glass surface^25^. In a recent work by Osnes et al., a stochastic strength model was developed to estimate the strength of glass panels subjected to arbitrary loading histories. This was done by utilizing FE simulation stress fields and by conducting virtual experiments on many glass panels^26^. This stochastic strength model was based on the research conducted by Yankelevsky^27^. Experiments on monolithic glass were presented for comparison purposes^13^.

In another study, a total of 15 LG specimens, consisting of PVB interlayer and annealed float glass plates, were experimentally tested under several pressure levels^28^. The tests result in varying post-fracture behavior due to the difference in the time and fracture initiation location within the different pressure levels. Higher-order elements with node-splitting capability were used instead of element erosion in the simulations of blast-loaded LG specimens. Both laminated and monolithic glass were studied. It was assumed that glass fractures occur upon reaching a deterministic failure criterion, although we acknowledge that probabilistic fracture strength should be used^10^. It was recommended that in the design of LG components, the glass stochastic fracture behavior should be considered. Additionally, another study explored the failure mechanisms of LG panels of varying thickness under blast impacts, employing various simulation approaches^29^. These included FE methods with element erosion or deletion based on specific failure criteria, mesh-free techniques, and hybrid combinations. Experimental validations confirmed the accuracy of these models in predicting maximum deflection responses.

In recent years, the use of sandwich structures has gained significant attention for blast-resistant applications due to their superior energy absorption and deformation mitigation capabilities compared to solid plate designs. Numerous studies have demonstrated that sandwich panels, particularly those incorporating honeycomb cores and fiber metal laminate or composite face sheets, offer remarkable blast resistance while maintaining a lower structural weight. For instance, Patel et al.^30^ performed a comparative study showing that sandwich plates exhibit considerably smaller back-face deflections and higher energy absorption than equivalent-mass stiffened and solid plates under explosive loads. Further, the incorporation of advanced composite materials such as CFRP in sandwich panels has been shown to significantly enhance blast mitigation performance while contributing to mass reduction^31,32^. While these findings have established the efficiency of sandwich panels, especially with uniform core structures, there remains a research gap in exploring hybrid core designs and novel configurations that synergize multiple energy dissipation mechanisms. Therefore, this study proposes a new approach by conducting a comprehensive numerical investigation into the effect of LG cross-sectional design on blast resistance, focusing on various glass thicknesses, and hybrid polymer interlayers, aiming to further improve blast resistance beyond the conventional sandwich panel designs.

The current study investigates the impact of the LG cross-section on blast performance. This work is built upon a previous study conducted on the static loading of LG and the effects of the layup configuration^33^. A high-fidelity numerical model using the Arbitrary Lagrangian–Eulerian three-dimensional analysis (ALE3D) multiphysics software is employed^34^, with blast load simulations verified using experimental data. The model was used to examine various glass and interlayer configurations, such as a glass double-layer, multi-layered glass with various arrangements, and double-layer glass with hybrid interlayers. A small optimization study was conducted varying the number of glass layers, the glass layer thicknesses, and the order of glass layers to optimize the LG panel performance during blast loadings.

## Dynamic material characteristics

**Fig. 1:**
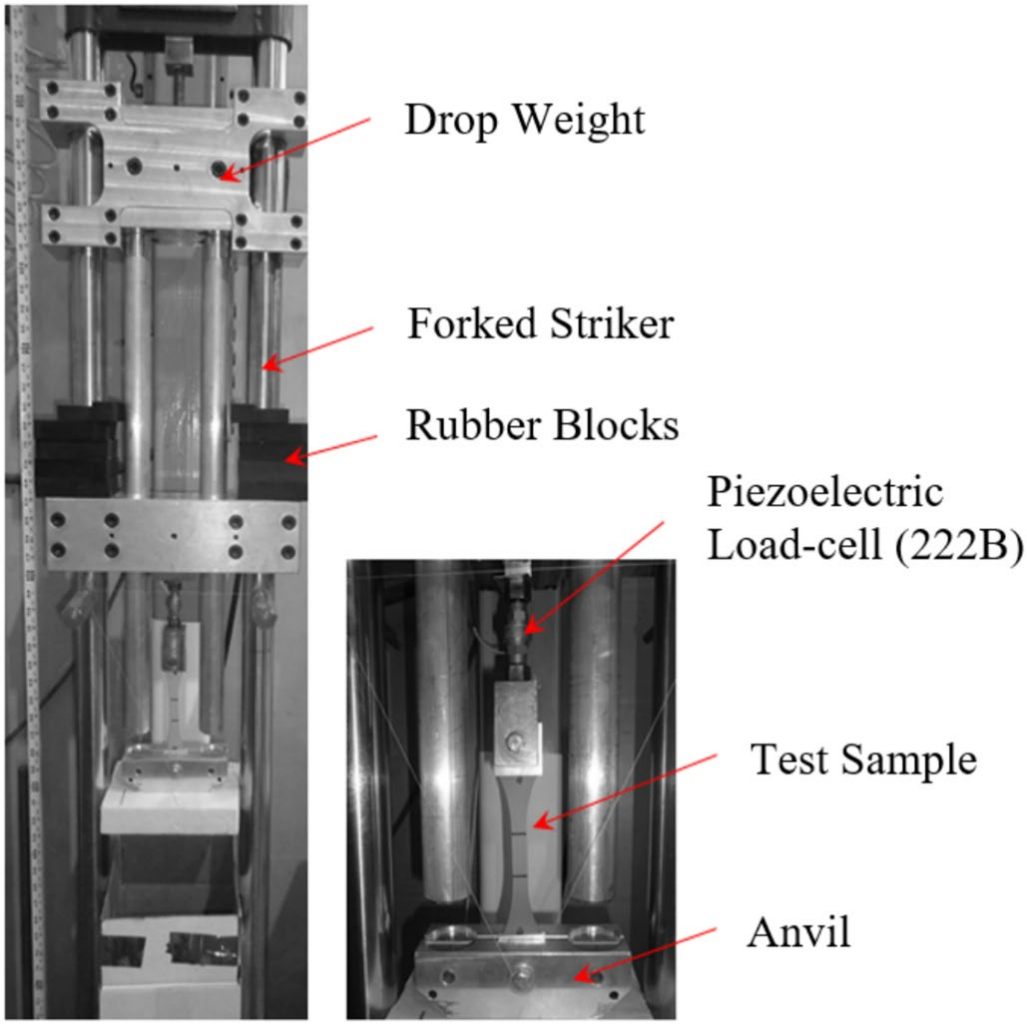
Drop weight machine.

The study used a drop-weight device (Fig. 1) to conduct dynamic tensile testing and achieve a high strain rate. These material characterization results are used to define the material properties for dynamic modeling. A piezoelectric load cell captured load data at a rate of 3000 data points per second to calculate the engineering stress. The elongation of specimens was measured with a high-speed camera recording at 3000 frames per second. To calculate the specimen’s total deflection, load cells with 112 N (250 pounds) capacity and an linear variable differential transformer (LVDT) were integrated into the apparatus. The specimen gauge length deflection was determined via a camera with high-resolution and specialized tracking video software. For a more detailed description of the dynamic testing setup, readers are encouraged to refer to our previously published work^35^.

In this section, the results of the material characterization will be presented and discussed. The analysis will include stress–strain relationships. A total of twenty specimens were tested, consisting of five samples for EVA, five samples for SG, five samples for PVB, and five TPU samples. Table 1 presents the results obtained from testing these polymeric materials at an average 45 s^−1^ strain rate. This strain rate falls within the range of strain rates observed both numerically and experimentally (20–75 s^−1^) during blast events^7,36^. The findings include the energy, which is obtained by calculating the area of the region below the stress–strain relation. These results are calculated to find the parameters of material modeling for the numerical modeling.

**Table 1:** Results of the high strain rate of 0.76 mm (0.03 in) processed specimens.

Material	Average strain rate (s^−1^)	Max. Strain (mm/mm)	Max. Engineering stress (MPa)	Energy (j/m^3^)
PVB	46.52	1.62	25.70	27.59
EVA	51.70	5.19	4.12	19.15
SG 6000	42.30	2.19	33.76	77.06
TPU	39.5	3.353	25.73	51.798

**Fig. 2:**
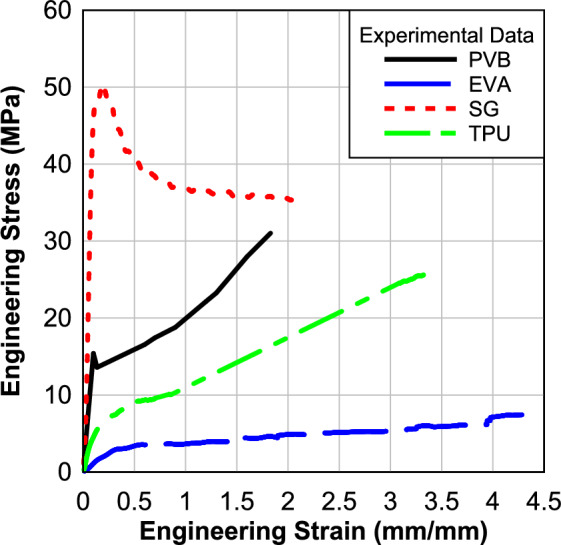
Engineering stress–strain response at strain rate 45^−1^.

Figure 2 displays the average engineering stress–strain relationships for the four material specimens (PVB, EVA, TPU, and SG) at a strain rate of 45 s^−1^. It can be observed that all specimens exhibit a similar pattern, characterized by a rapid increase in engineering stress initially, followed by a relatively smaller increase in stress values as the strain continues to increase. Only the SG showed a softening performance after the peak. This consistent pattern suggests a similar mechanical response and behavior among the tested materials under the specified strain rate. The results were discussed in detail in our previous publication^35,37^.

## Numerical model

In this section, the numerical modeling will be described. The description will include the material models, the element types, the solution options, and the loading mechanism.

### Model geometries

An explicit nonlinear FE model was developed to predict the displacement response and failure modes of the LG windows. The model components, material models, model loading, and solution will be discussed. The model was built using the ALE3D explicit FE code and was validated using experimental results.

**Fig. 3:**
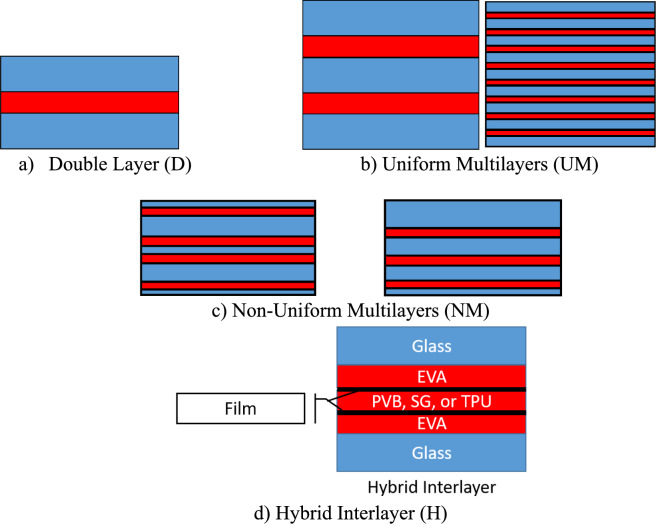
Example LG configurations investigated in this study.

To study the different parameters that affect the performance of the LG panels, a numerical parametric study was performed using the validated model. The study will focus on the full-scale panels, so the size of these panels is 965.2 mm × 1676.4 mm with 30 mm rubber support. The matrix includes 6 different LG panel section types that will be modeled to predict the strength and response of the panels, as seen in Fig. 3. These geometries will be used to generate 26 different cases with different material and geometric characteristics as shown in Table 2. The 26 cases were solved under different blast load histories.

**Table 2:**
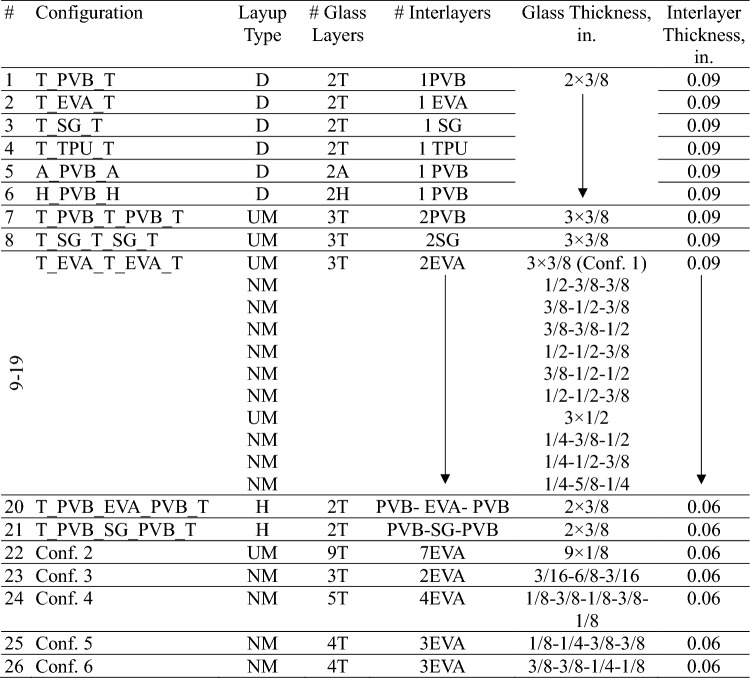
Laminated glass designs.

*D* Double layer; *UM* Uniform layers; *NM* Non-uniform layers; *H* Hybrid Interlayer; *T* Tempered glass; *A* Annealed glass; *H* Heat strengthened glass.

### ALE3D simulation setup

**Fig. 4:**
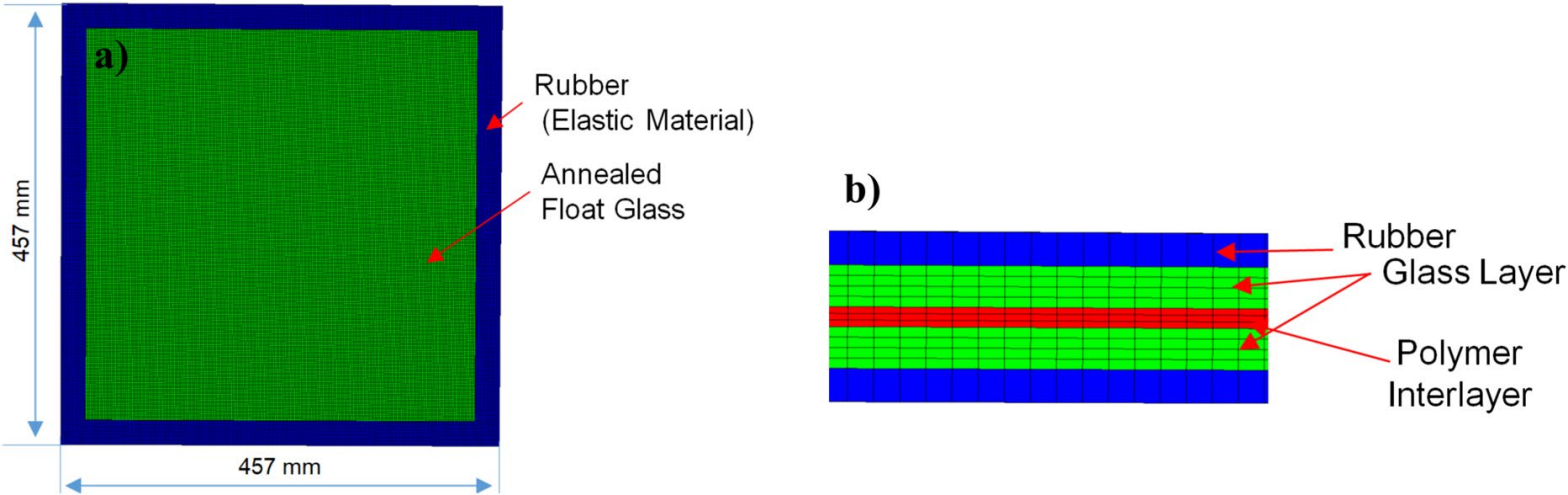
Explicit finite element model: (**a**) full model window panel and glazing rubber, and (**b**) cross-section of the window panel.

The blast analysis was conducted using the ALE3D explicit FE code. ALE3D utilizes arbitrary Lagrangian–Eulerian techniques and is suitable for many multiphysics problems, including blast-type events. For this work, a Lagrangian formulation with element deletion is used to allow crack propagation within the glass. The model consists of multilayers of glass and polymer interlayers with a rubber strip (1/8-in. thick) along the outer edge on the top and bottom of the LG. The rubber strip mimics the clamping boundary conditions of the experiment. All elements in the model were 3-D hexahedral elements with reduced integration. Figure 4 illustrates the explicit FE model in ALE3D.

The thickness of the glass and interlayer depends on the layup being studied, with each layer consisting of two elements through the thickness with a cross-section element size of 2.9 mm by 2.9 mm. This element size was chosen after a convergence study and was shown to reliably capture the deflection-time response and crack patterns. For this work, a fixed interface between layers was used. The top surface of the rubber strip is fixed in all directions to mimic clamped boundary conditions. A pressure–time history emulating a blast wave is applied to a node set consisting of one side of the LG panel. The maximum displacement of the interlayer is tracked throughout the simulation to produce pressure versus displacement graphs.

### Material models

In this section, the material behaviors will be discussed in detail. Glass is well known as a brittle material that has linear stress–strain relation up to failure. Several models can be used to simulate the glass, such as simple isotropic damage models^38,39^ or advanced probabilistic damage models. Johnson-Holmquist is an available material model in several commercial software, and it has proven efficiency in predicting the experimental response of LG^33^. Due to the unavailability of the probabilistic models in ALE3D, Johnson-Holmquist was used to simulate the glass in the current study^40,41^. The JH-2 model is a pressure and strain rate-dependent model with progressive damage that has been widely used to study fracture events in brittle materials like ceramics and glasses. The JH-2 model acts much like a standard brittle fracture model with a linear loading up to the fracture point. JH-2 implements progressive damage, which reduces the dramatic stress drop of brittle materials. Once fully damaged, the material will only resist hydrostatic crushing.

The normalized von Mises stress is defined as intact (${\upsigma }_{i}^{*}$) and damaged (${\upsigma }_{f}^{*}$) material:

1 $${\upsigma }^{*}=\frac{\upsigma }{{\upsigma }_{hel}}={\upsigma }_{i}^{*} - D({\upsigma }_{i}^{*}-{\upsigma }_{f}^{*})$$

where.

2 $${\sigma_i}\, = \,A{{(^*}\, + \,{T^*})^N}(1\, + \,C\ln )\varepsilon$$

3 $${\sigma_f}\, = \,B{\left( {P^*} \right)^M}(1\, + \,C\ln )\varepsilon$$

and ${\dot{\upepsilon }}^{*}$ is the dimensionless strain rate equal to $\dot{\upepsilon }/{\dot{\upepsilon }}_{0}$, D is the damage, A, B, C, and N are material constants, and P^*^ and T^*^ are the normalized pressure and max tensile pressure. The damage rate is defined as a function of strain rate by:

4 $$\dot{D}=\frac{{\dot{\upepsilon }}_{p}}{{\upepsilon }_{p}^{f}}$$

where the plastic strain to fracture is defined by:

5 $${\upepsilon }_{p}^{f}={D}_{1}{\left({P}^{*}+{T}^{*}\right)}^{{D}_{2}}$$

where ${D}_{1}$ and ${D}_{2}$ are material damage constants. In this work, to add some stochastic variation to the fracture of the glass, the damage constants ${D}_{1}$ and ${D}_{2}$ are initialized around a random mean with a maximum perturbation of 0.02 and 0.2, respectively. Finally, an equation of state (EOS) relationship is defined by:

6 $$P={K}_{1}\upmu +{K}_{2}{\upmu }^{2}+{K}_{3}{\upmu }^{3}+\Delta {\text{P}}_{\text{bulk}}$$

where $\Delta {P}_{bulk}$ is a bulking pressure term that accumulates linearly with damage, μ is the volumetric strain, and K_1_, K_2_, and K_3_ are material constants.

The model requires 15 parameters: two for the linear elastic properties (shear and bulk modulus), nine to define pressure and strain rate-dependent strength, and two each to define the damage and the EOS. Constants from Table 3 list the values used for this material model for the 3 different glasses. Parameters from the original JH-2 work for float glass were used apart from the tensile strength, which is dependent on the glass type. The model can capture fractures of annealed, heat-treated, and tempered glass by changing the tensile strength of the material and has been used successfully to capture fractures in float glass^42,43^.

**Table 3:** Material Properties of glass used in the explicit analysis.

Parameter	Value		
	Annealed	Tempered	HS*
1. Bulk modulus	45 GPa	45 GPa	45 GPa
2. Shear modulus	30 GPa	30 GPa	30 GPa
3. Jh2a: intact strength factor	0.93	0.93	0.93
4. Jh2b: fractured strength factor	0.35	0.35	0.35
5. Jh2c: strain rate factor	0.003	0.003	0.003
6. Jh2n: intact strength exponent	0.77	0.77	0.77
7. Jh2m: fractured strength exponent	0.4	0.4	0.4
8. Jh2ref: reference strain rate	1	1	1
9. JH2t: tensile strength	30 MPa	110 MPa	53 MPa
10. Jh2sfmax: maximum normalized fracture strength	0.5	0.5	0.5
11. Jh2hel: hugoniot elastic limit	5.95 GPa	5.95 GPa	5.95 GPa
12. Jh2k1: pressure coefficient	45.4 GPa	45.4 GPa	45.4 GPa
13. Jh2k2: pressure coefficient	− 138 GPa	− 138 GPa	− 138 GPa
14. Jh2k3: pressure coefficient	290 GPa	290 GPa	290 GPa
15. Jh2d1: damage coefficient	0.043	0.043	0.043
16. Jh2d2: damage coefficient	0.85	0.85	0.85

*Heat-strengthened glass.

For the polymer interlayer materials, the stress–strain relation evaluated from the experimental testing was used to select the best-fitting material models. The material model parameters for all the interlayers are found in Table 4. For EVA and TPU, the stress–strain response is best captured by a hyperelastic model. Thus, the Mooney-Rivlin hyperplastic model of 2nd order was used, which needs two calibration parameters, i.e., A and B. For SG, a bilinear elastoplastic material model was used because the material exhibits clear yielding and softening behaviors. This model needs parameters such as Young’s modulus, yield strength, and hardening modulus. For the PVB, the Johnson–Cook plasticity model was used. The model needs several parameters, i.e., jc_a, jc_b, and jc_n. Strain-rate dependence was not considered, and the polymeric materials were calibrated to best capture their stress–strain behavior at a strain rate of 45 s^−1^, which is a typical strain rate achieved during a blast load. The maximum Von Mises failure criteria were used for all the interlayer materials (Table 4) as an erosion criterion, although most LG configurations can be evaluated long before the interlayer begins to tear. It is worth noting that the simplified models used for the polymer interlayers will not capture the strain rate effects and will not properly capture the load-unload behavior of the materials. However, these effects on the maximum deflection are expected to be small as the maximum deflection is dominated by the initial stiffness and glass fracture strength.

**Table 4:** Material Model Parameters for Polymer Interlayers.

Mooney Rivlin (Viscoelastic)					
	Density (g/cc)	Poisson	A (MPa)	B (MPa)	Failure: von Mises True Stress (MPa)
EVA	940	0.48	0.220	0.206	40
TPU	1100	0.48	0.310	0.0	110

Bilinear Elastic–Plastic for Ionomer						
	Density (g/cc)	Poisson	Young’s Modulus (MPa)	Yield Stress (MPa)	Hardening Modulus (MPa)	Failure: von Mises True Stress (MPa)
SG	950	0.45	500	50	51	107

Johnson–Cook Elastic–Plastic							
	Density (g/cc)	Poisson	Young’s Modulus (MPa)	jc_a (MPa)	jc_b (MPa)	jc_n (MPa)	Failure: von Mises True Stress (MPa)
PVB	950	0.45	165	16.5	301	2.95	90
TPU	1100	0.42	95	8	8000	6.5	110

**Fig. 5:**
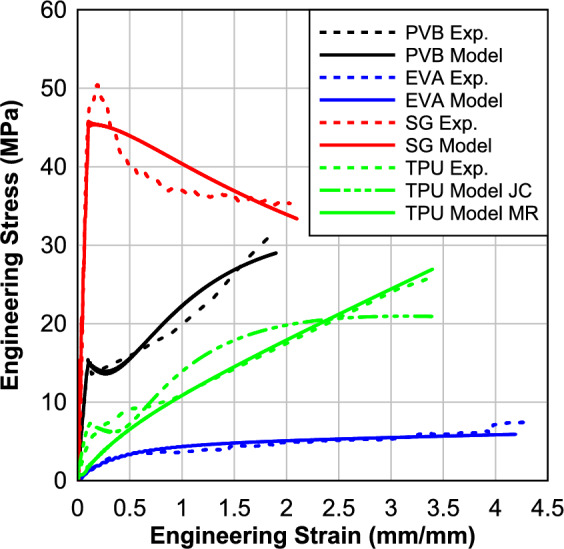
Comparison between the experimental test data and the proposed models.

Figure 5 shows a comparison between the experimental stress–strain curve and the analytical ones used for the FE analysis.

### Loading and solution parameters

**Fig. 6:**
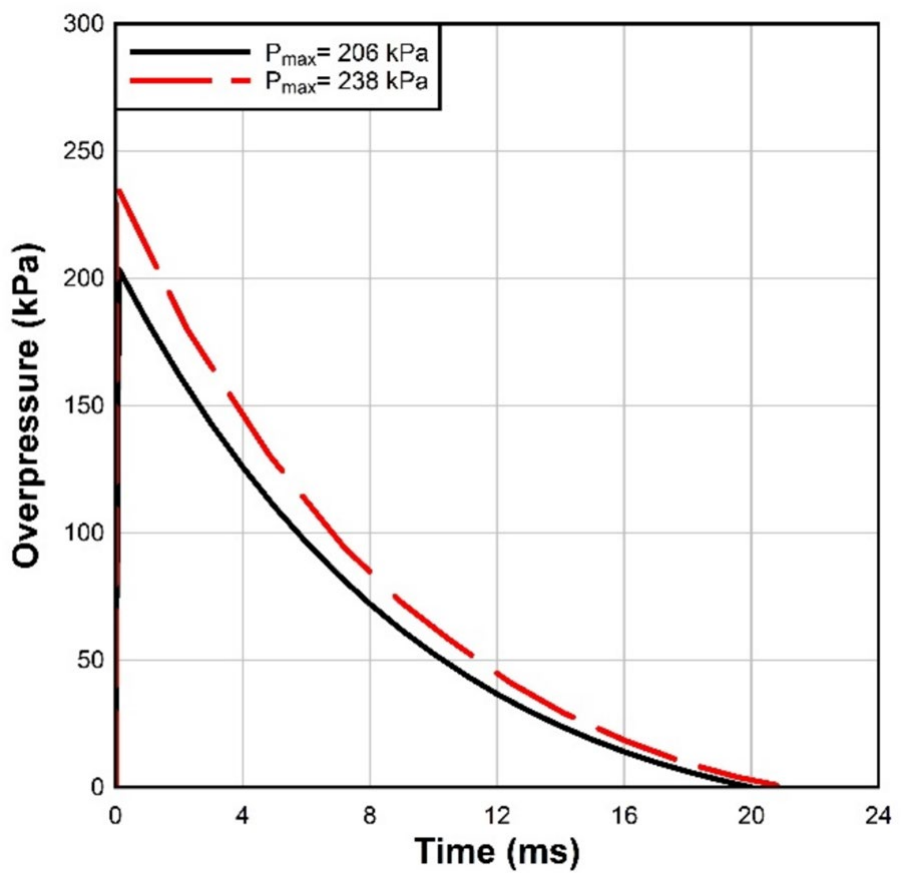
Reflected pressure time history.

For validation against experimental data done by Osnes et al.^10^, the specimen is loaded by the reflected overpressure, represented by the fitted Friedlander. Two pressure histories were used in the analysis as shown in Fig. 6. The pressure was applied to the outer surface of the glass panel.

The FE mesh of the LG system under loading is shown in Fig. 4 at two stages of displacement. The mass of eroded elements was redistributed to neighboring nodes. Data is output every 1e^-5^ s, and a smoothing window of 300 is used when plotting the data.

In addition, Kranzer et al.^44^ highlighted the critical importance of using accurate loading conditions in simulations of dynamic responses. Instead of assuming a triangular loading profile, which is commonly adopted in the literature, the ALE3D^34^ model employed the actual pressure–time history obtained from the shock tube test as the dynamic loading. This approach ensured a more realistic representation of the loading conditions, improving the accuracy of the simulation results.

## Results and discussions

In this section, the numerical results will be presented and discussed. The discussion will encompass results such as numerical validation, failure modes, and the effects of different parameters. The dynamic performance, on the other hand, will be highlighted through the utilization of numerical modeling. To validate the numerical modeling, dynamic experimental data from the literature was employed.

### Validation of finite element model

The validation of the numerical models was carried out using data from two publicly accessible field experiments. The initial FE validation relied on experimental findings reported by Osnes et al.^10^, while the subsequent validation employed data obtained from the experiments conducted by Kranzer et al.^44^.

#### Osnes validation

**Fig. 7:**
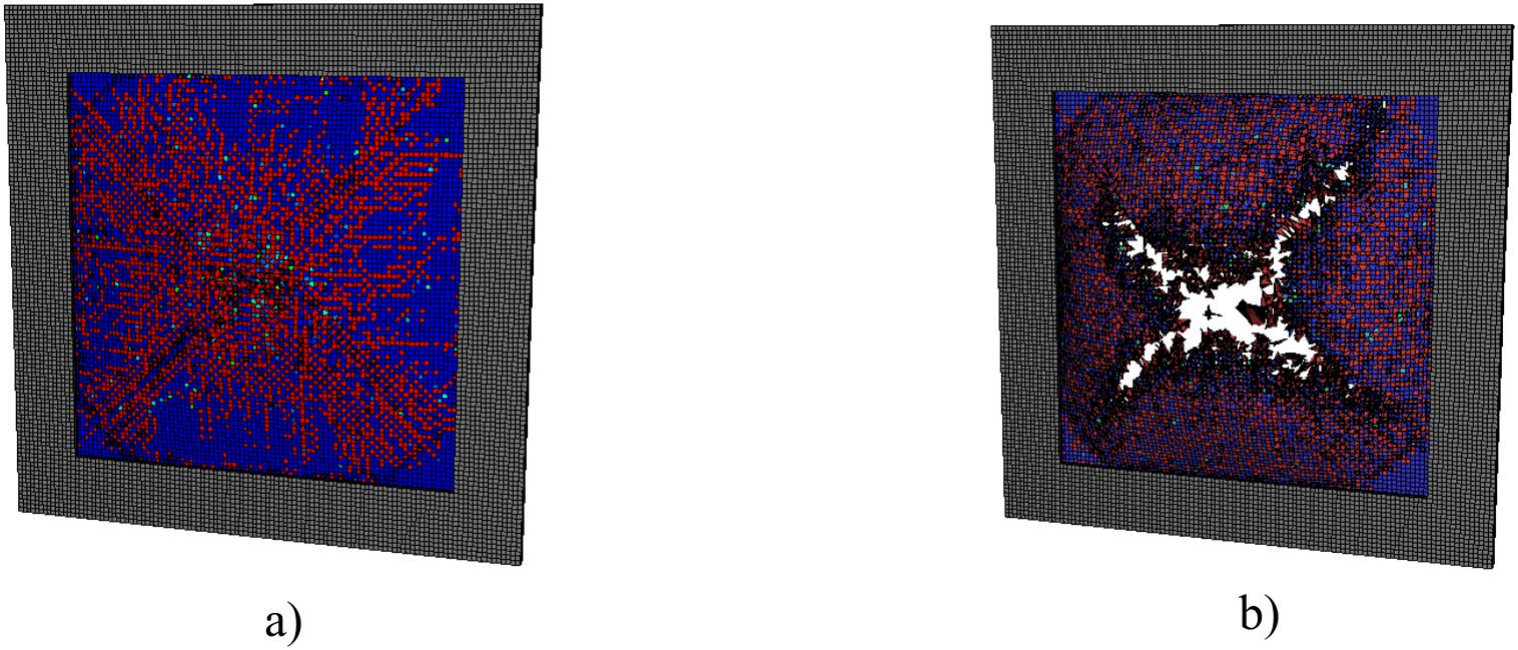
Examples of final explicit model color by total displacement.

For validation purposes, an experimental study done by Osnes et al. was modeled^10^. It consists of an LG panel subjected to lateral blast pressure resulting in fracture, as seen in Fig. 7a, b. The glass specimen has dimensions of 400 mm × 400 mm, while the loaded area is 300 mm × 300 mm. The glass consists of two 4 mm annealed glass layers and one 3.8 mm PVB polymer interlayer. For more details on the experimental setup, we refer to Osnes et al.^10^. Neoprene rubber strips with a thickness of 4 mm are glued to the clamping plates and positioned on each side of the glass. The specimen is clamped between two 25 mm thick aluminum plates. All elements in the model were 3D hexahedral elements. The window panel is composed of three layers (two outer glass layers and one polymer interlayer), with each layer consisting of two elements through the thickness with a cross-section element size of 2.3 × 2.3 mm. For this work, a fixed interface between layers was used.

**Fig. 8:**
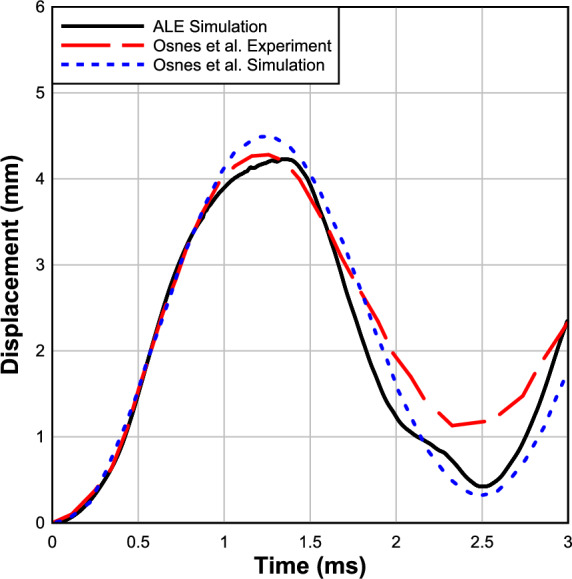
Comparison between numerical and experimental results of Osnes et al. ^10^ (pressure = 205 kPa and impulse = 1379.9 kPa ms).

Osnes et al. provided precise pressure–time history curves for the test^10^, which were then exported to ALE3D^34^ with a maximum pressure and impulse of 205.3 kPa and 1379.9 kPa-ms. Consequently, the ALE3D model utilized the actual load-time history for dynamic loading, deviating from the commonly assumed triangular loading often found in the literature. As shown in Fig. 8, the numerical and experimental results for maximum deflections of glass panels agreed satisfactorily. Table 5 shows the comparisons between the experimental data and ALE3D findings for the test done by Osnes et al.

**Table 5:** Deflection comparisons of the verification window.

Sample	Maximum dynamic deflection (mm)
Experiment	4.28
FE Analysis	4.23
% Difference	1.18

Furthermore, as shown in Fig. 8, the ALE3D FE predictions showed better conformity to the experiment results and matched the measured dynamic deflection during the early portion of the response until the peak deflection. In the current study, the difference between the peak deflection of experimental and numerical results is less than 1.2%.

#### Kranzer validation

The numerical models were validated using experimental data obtained from a shock tube test by Kranzer et al.^44^. The tested LG panel measured 1,100 mm in width and 900 mm in height, comprising two annealed glass panes, each 7.5 mm thick, bonded by a 1.5 mm PVB polymeric interlayer. To secure the LG panel within the frame, 50.8 mm wide rubber bites were employed. During the test, the LG panel was subjected to a dynamic pressure of 58 kPa and an impulse of 98.6 kPa-ms.

**Fig. 9:**
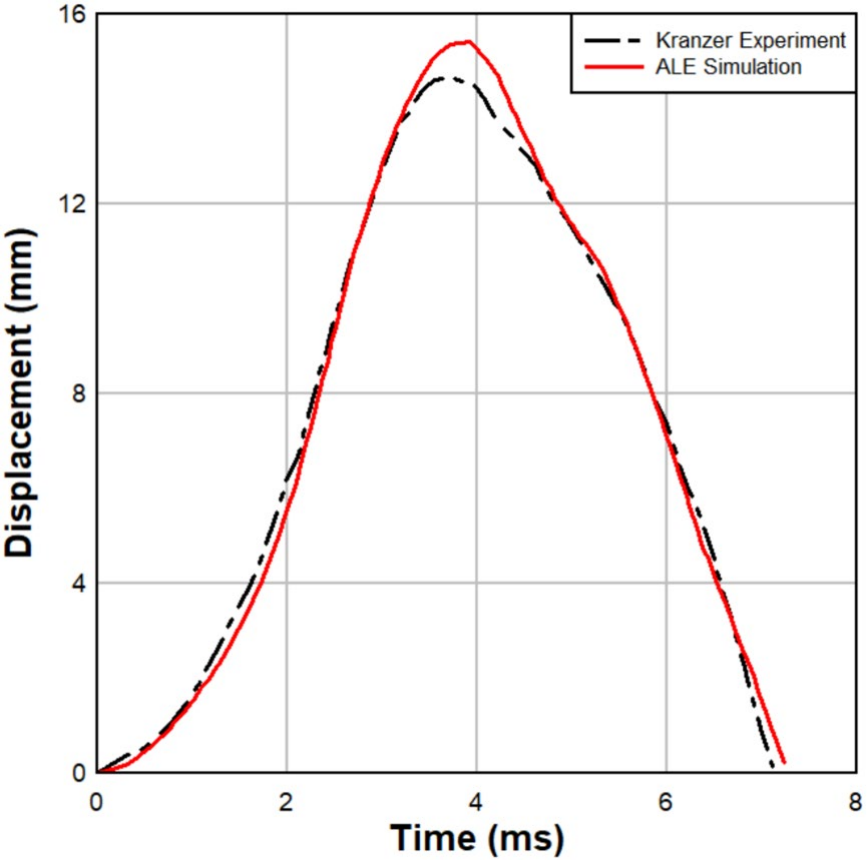
Comparison between numerical and experimental results of Kranzer ^44^.

The experimental pressure–time history obtained from the shock tube test was imported into ALE3D^34^ to simulate the dynamic response of the LG specimen. Figure 9 illustrates the comparison between the ALE3D and experimental responses, highlighting close agreement, particularly during the initial phase of the dynamic response and up to the peak deflection and extending beyond the peak deflection. The midspan deflection observed experimentally was approximately 14.7 mm, while the FE model predicted a deflection of about 15.4 mm. This represents a difference of 4.7%, demonstrating a satisfactory correlation between the experimental and numerical results. The results indicate that the ALE3D predictions align well with the experimental data, particularly in capturing the early stages of the deflection response even after the peak deflection.

### Effect of interlayer type

**Fig. 10:**
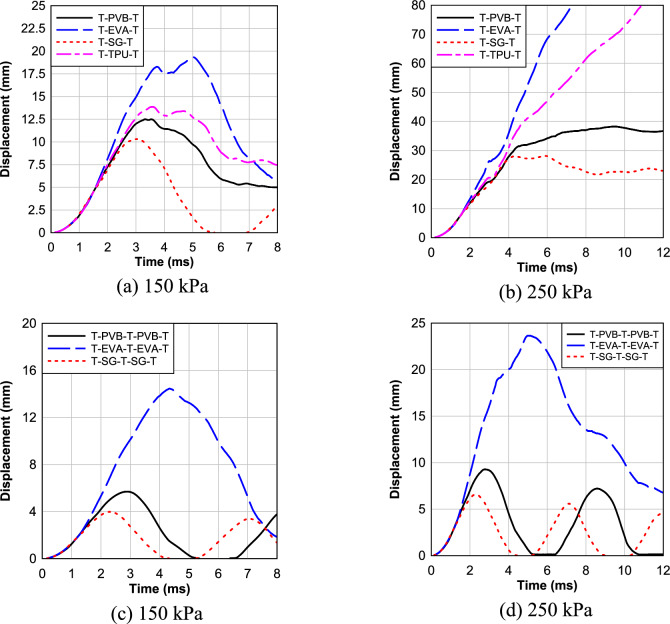
Effect of Interlayer Type.

The effects of interlayer types on the LG panels under a high and low blast load are investigated first. Figure 10a depicts the response of four different LG panels, each incorporating distinct interlayers (PVB, EVA, SG, TPU), subjected to a blast pressure of 150 kPa and an impulse of 1150.2 kPa-ms. Notably, the panel with an SG interlayer exhibited a maximum deflection of 10.3 mm at the center. Remarkably, this configuration displayed less damage in the interlayer compared to other samples, as depicted in Fig. 10a. In contrast, panels with PVB and TPU interlayers demonstrated higher deflections at the center, measuring 12.49 mm and 13.86 mm, respectively. Notably, the specimen with an EVA interlayer displayed the highest deflection at the center, measuring 19.34 mm.

Moving on to Fig. 10b, which explores the response of the same LG panels under a higher blast pressure of 250 kPa and impulse of 1939.6 kPa-ms, the SG interlayer again showcased favorable characteristics. The panel with SG interlayer exhibited the lowest maximum deflection of 28.15 mm at the center, with less damage observed in the interlayer compared to other samples. Conversely, specimens with PVB and TPU interlayers displayed higher deflections at the center, measuring 38.24 mm and 97.71 mm, respectively. Notably, the specimen with an EVA interlayer suffered the most severe consequences, with a deflection measuring 150.68 mm, leading to complete panel failure and substantial damage in the interlayers.

Moving on to Fig. 10c, three different multilayer LG panels (three glass layers and two layers of polymeric interlayer) with PVB, EVA, and SG interlayers are studied under a blast pressure of 150 kPa. The panel with the SG interlayer again demonstrated superior performance, exhibiting the lowest maximum deflection of 3.98 mm at the center, with less damage observed in the interlayer. In contrast, panels with PVB and EVA interlayers displayed higher deflections at the center, measuring 5.7 mm and 14.45 mm, respectively. As before, the specimen with an EVA interlayer experienced panel failure, accompanied by significant damage in the interlayers.

Lastly, Fig. 10d investigates the performance of the same multilayer LG panels under a higher blast pressure of 250 kPa. The SG interlayer once again demonstrated favorable characteristics, displaying the lowest maximum deflection of 6.59 mm at the center, with less damage observed in the interlayer compared to other samples. Conversely, panels with PVB and EVA interlayers showed higher deflections at the center, measuring 9.27 mm and 23.65 mm, respectively. Again, the specimen with an EVA interlayer experienced the highest deflections, resulting in panel failure and substantial damage in the interlayers.

Overall, the effect highlights the significant impact of interlayer types on the blast response of LG panels. The SG interlayer consistently outperforms PVB, EVA, and TPU counterparts, exhibiting deflections consistently within a range of 15–40% lower than other interlayers across different blast pressures. Adding a third tempered glass layer significantly reduces peak deflection compared to the double-layer systems. These findings underscore the crucial role of glass configuration and interlayer material in enhancing blast resistance.

**Fig. 11:**
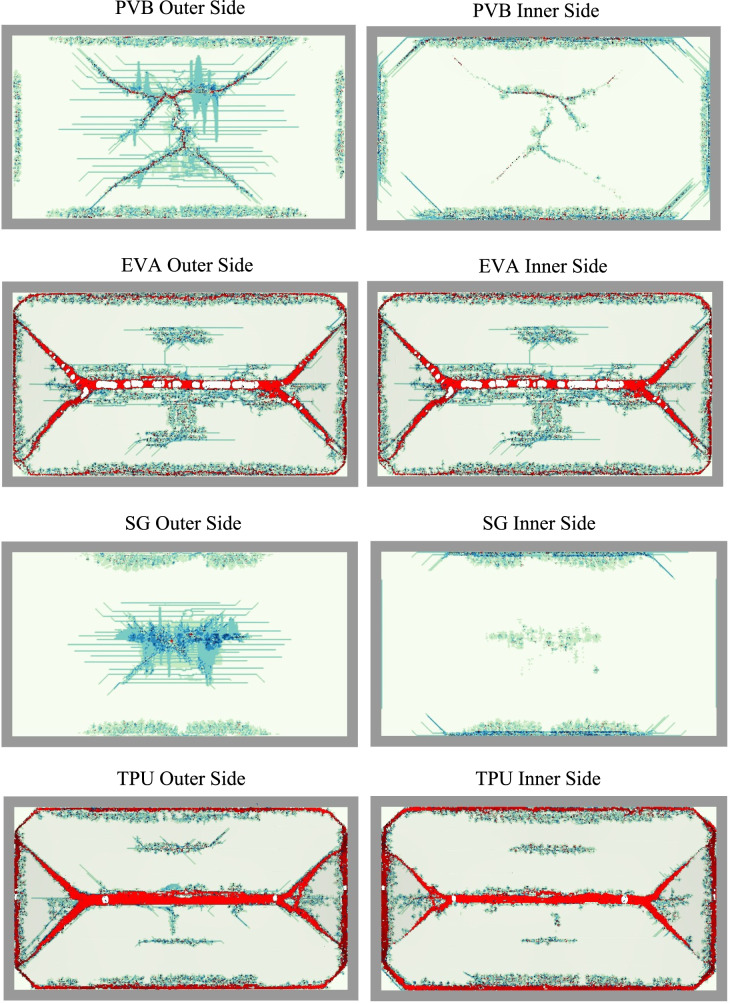
Fracture patterns for LG systems consisting of PVB, EVA, SG, and TPU interlayers subjected to a 250 kPa max blast pressure. Grey represents the outer rubber strip clamping the LG, red represents the interlayer material, and the blue-green color represents the JH-2 damage parameter for the glass.

Additionally, the various interlayer and layup configurations create different fracture patterns in the glass layers. Figure 11 illustrates the various fracture patterns for the double-layer LG systems with different interlayers. LG with EVA and TPU interlayers both undergo catastrophic failure with the glass fracturing into four large sections. Interlayer tearing is also observed, particularly in the EVA system. SG and PVB interlayer systems have some observed cracking, but the fracture pattern is more diffuse compared to the EVA and TPU, which failed catastrophically. LG with SG interlayer only experiences the onset of fracture within the inner glass. The other LG configuration in this study failed with similar failure patterns. Most notably, LG systems that catastrophically failed all showed similar fracture patterns as the EVA and TPU systems.

### Effect of interlayer hybridization

**Fig. 12:**
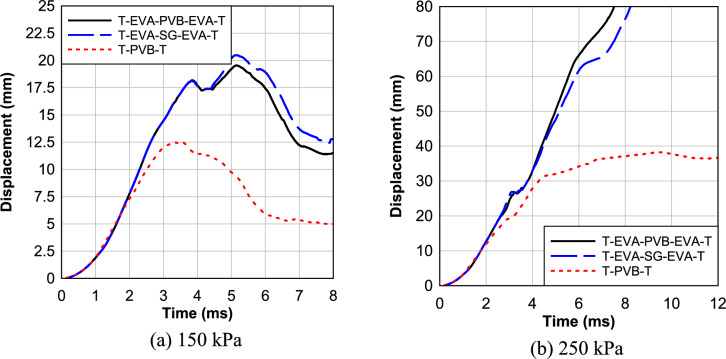
Effect of interlayer hybridization.

In the investigation of the effects of interlayer hybridization on the LG panels under blast loads, Fig. 12 is dedicated to studying the response of two different LG panels, each incorporating distinct hybrid interlayer combinations (T_PVB_EVA_PVB_T and T_PVB_SG_PVB_T), subjected to a blast pressure of 150 and 250 kPa compared to panels with pure PVB interlayer. Hybrid interlayers underperformed the equivalent thickness PVB LG panel under both the low and high blast loads. Possibly due to unbalanced load sharing between a stiffer middle interlayer and the more compliant EVA outer membrane. A better setup would be as thick as possible mid-layer (SG) and as thin as possible outer EVA layers to aid in glass adhesion.

### Effect of glass type

**Fig. 13:**
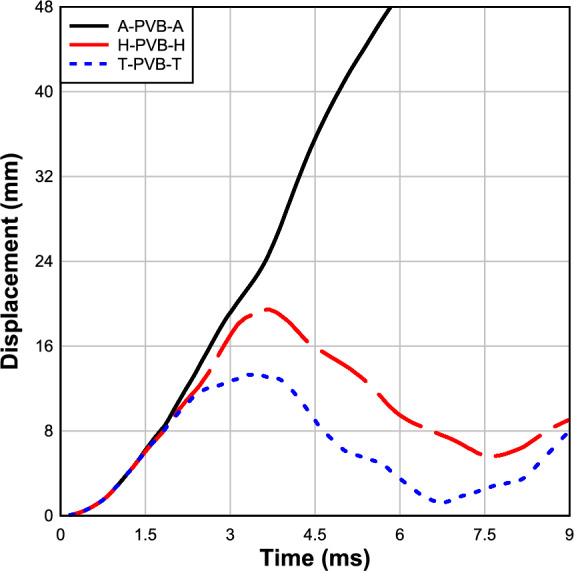
Effect of glass type.

In the investigation of the effects of glass types on the blast resistance of LG panels, Fig. 13 examines the response of LG panels with three distinct glass types (annealed, heat strengthened, tempered) under a blast pressure of 150 kPa. Notably, the panel featuring tempered glass exhibited superior performance, displaying a maximum deflection of 12.5 mm at the center with less permanent deflection observed in the system compared to other samples, as illustrated in Fig. 13. Conversely, panels with heat-strengthened glass showcased higher deflections at the center, measuring 19 mm. Notably, the specimen incorporating annealed glass experienced the highest deflections at the center, measuring 100 mm, leading to panel failure and substantial permanent deflection, as depicted in Fig. 13. This marked difference in performance can be attributed to the inherent characteristics of tempered glass, which boasts a high tensile stress capacity, four times that of annealed glass and twice that of heat-strengthened glass.

In general, tempered glass demonstrates superior blast resistance with deflections consistently lower than other glasses, showing a notable percentage reduction of approximately 80% compared to annealed glass and 34% compared to heat-strengthened glass in the numerical modeling results. These findings underscore the substantial impact of glass type on enhancing structural integrity under blast loading conditions.

### Effect of glass layer configuration

**Fig. 14:**
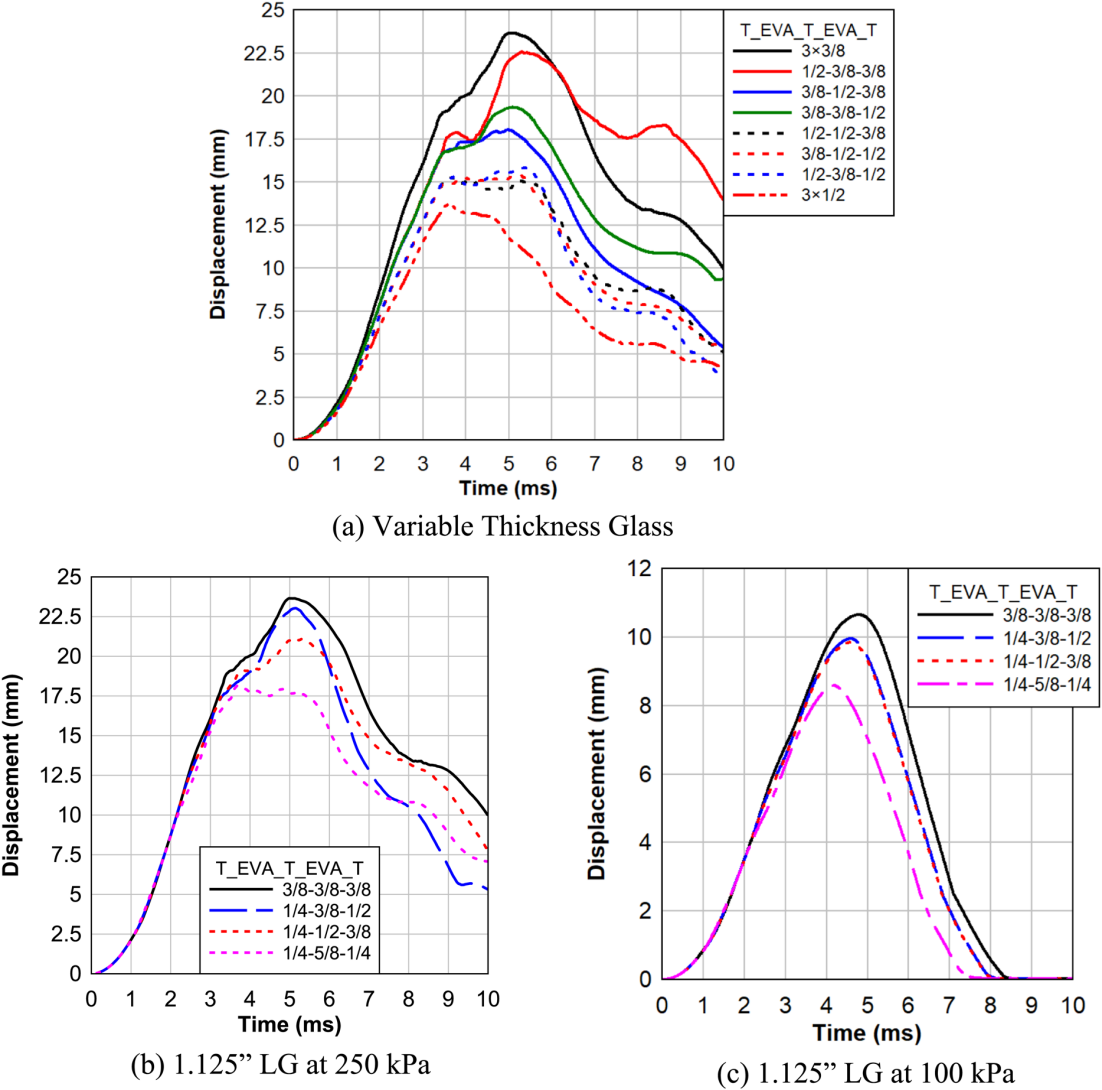
Effect of Glass Layer Configuration.

In this study, all LG panels consist of multiple layers, comprising three layers of tempered glass and two layers of EVA polymeric material. EVA was chosen as the interlayer as it adds the least resistance to the system, highlighting the specific performance of the glass configuration. The results presented in Fig. 14a highlight the significant impact of glass layer configuration on the response of multi-layer LG panels, particularly when replacing a 3/8″ panel with a 1/2″ thick panel. It is evident that an overall transition to 1/2″ thickness across all layers leads to a substantial reduction in maximum deflection, decreasing from 23.65 mm in the 3/8″ configuration to 13.71 mm, which corresponds to a remarkable 73% decrease. Moreover, when isolating the impact of layer thickness changes, focusing on the loaded (inner) layer proves more effective in minimizing deflection, with a thickness of 1/2″ resulting in a deflection of 19.34 mm compared to 22.56 mm for the non-loaded (outer) layer. Surprisingly, changing the mid-layer to 1/2″ thickness yields even better performance, with a deflection of 18.02 mm. Furthermore, exploring the effect of replacing two layers with 1/2″ thickness, it is observed that the 1/2″ Outer + 1/2″ Mid configuration exhibits a slight advantage, offering a 1–3% reduction in deflection compared to 1/2″ Mid + 1/2″ Inner and 1/2″ Outer + 1/2″ Inner configurations.

Moving on to Fig. 14b, which investigates the response of LG panels with a total thickness of 1 1/8″ under 250 kPa blast loads with different glass layer configurations and thicknesses, distinct trends emerge. The configuration of three layers with the same thickness (3/8″) results in the highest maximum deflection (23.65 mm) and more severe damage to the LG panel. However, configurations such as 1/4″-1/2″-3/8″ and 1/4″-5/8″-1/4″ demonstrate reduced deflections of 21.09 mm and 18.19 mm, respectively. Notably, the latter configuration with a thicker mid-layer exhibits the least deflection at the center of the specimens, surpassing the other configurations by 15–30%. This emphasizes the crucial role of layer thickness distribution in enhancing blast resistance.

Figure 14c extends the analysis to LG panels under 100 kPa blast loads, reinforcing the trends observed in Fig. 14b. The configuration of three layers with the same thickness (3/8″) yields the highest maximum deflection (10.64 mm). Contrarily, configurations like 1/4″-1/2″-3/8″ and 1/4″-5/8″-1/4″ showcase reduced deflections of 9.84 mm and 8.58 mm, respectively. Once again, the configuration with a thicker mid-layer proves to be the most effective in minimizing deflection, exhibiting a 15–24% advantage over other configurations. These findings underscore the critical influence of glass layer configuration on the blast response of multi-layer LG panels, providing valuable insights for optimizing their performance in real-world scenarios.

In summary, the study on multi-layer LG panels reveals a substantial influence of glass layer configuration on panel response. Transitioning to an all 1/2″ thickness configuration significantly reduces maximum deflection by 73%. Under 250 kPa blast loads, configurations with varied layer thicknesses demonstrate up to a 30% reduction in deflection compared to uniform thickness configurations. Similarly, under 100 kPa blast loads, varying thickness configurations lead to a 24% reduction in deflection, highlighting the importance of tailored layer configurations for optimizing panel performance.

### Finding optimal layup

The exploration of optimal layups for multi-layer LG panels under blast loads, while maintaining a constant total glass thickness of 1 1/8″, involved the investigation of six different configurations through numerical modeling. In the first configuration, three constant glass layers with a thickness of 3/8″ each were analyzed as shown previously. The second configuration consisted of nine constant glass layers, each with a thickness of 1/8″. The third configuration introduced a variation with the middle layer at 6/8″ and the outer layers at 3/16″ each. The fourth configuration involved five alternating glass layers, starting with the inner layer at 1/8″ and sequentially increasing to 3/8″ in the middle layer and back to 1/8″ in the outer layer. The fifth configuration comprised four in-to-out gradient layers, starting with the inner layer at 1/8″ and gradually increasing to 3/8″ in the outer layer. Finally, the sixth configuration involved four out-to-in gradient layers, starting with the inner layer at 3/8″ and gradually decreasing to 1/8″ in the outer layer.

**Fig. 15:**
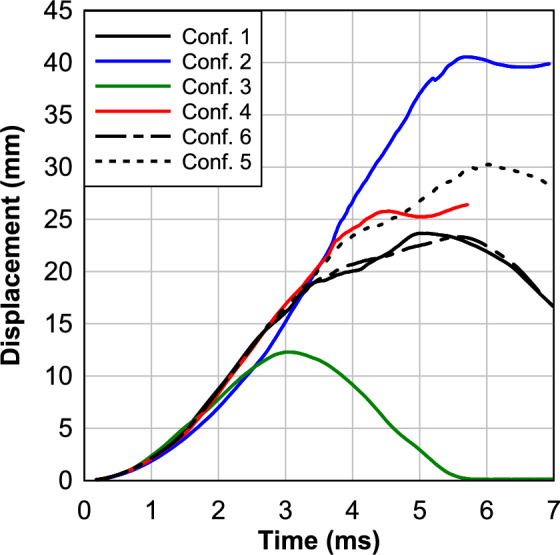
Response of different configurations.

From Fig. 15, it is evident that configurations 1, 2, and 3 exhibit the maximum mid-span deflections, measuring 23.65 mm, 40.54 mm, and 12.28 mm, respectively. Notably, Configuration 2 displays severe damage in the panel under blast loads compared to the other configurations. On the other hand, Configurations 4, 5, and 6 showcase mid-span deflections of 26.41 mm, 30.25 mm, and 23.32 mm, respectively. Significantly, Configuration 6, characterized by an out-to-in gradient, demonstrates lower deflection and superior performance compared to Configuration 4 (alternating glass) and Configuration 5 (in-to-out gradient) by 13% and 30%, respectively.

**Fig. 16:**
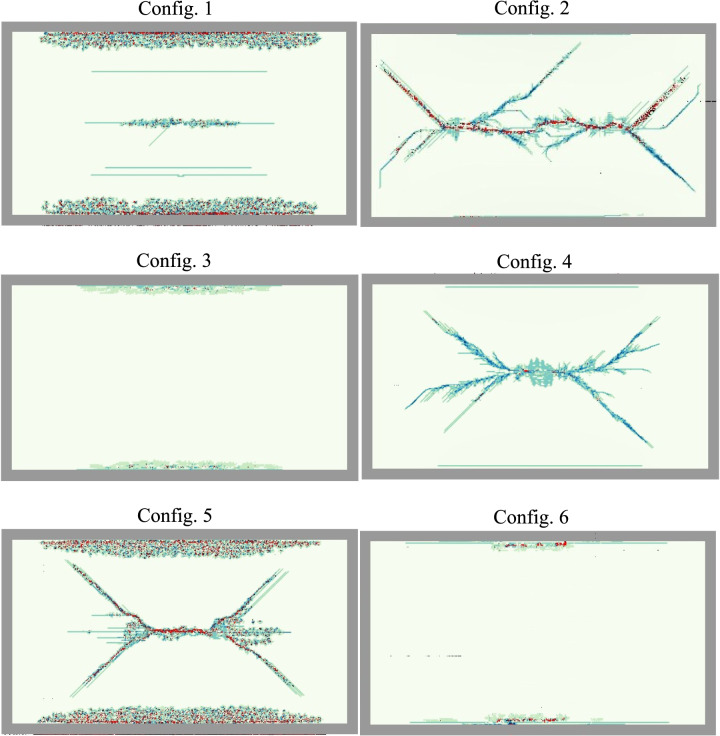
Outer glass crack patterns of the 6 layup configurations subjected to a 250 kPa max blast pressure. Grey represents the outer rubber strip clamping the LG, red represents the interlayer material, and the blue-green color represents the JH-2 damage parameter for the glass.

Figure 16 illustrates the crack patterns of the outer glass for various configurations. The severity of glass cracking follows closely with the relative max deflection performance of each configuration. Configurations 2, 4, and 5 perform the worst and display significant glass cracking, but no interlayer failure. Configurations 2 and 4 also display unique crack patterns due to the thin (1/8″) outer glass thickness. The thinner outer glass promotes higher levels of crack branching compared to other configurations, and Configuration 2 is very close to catastrophic failure as seen in Fig. 11. Configurations 1 and 6 exhibit some minor damage starting around the edges and at the center line of the glass. The top performer, Configuration 3, exhibits little damage and no residual deflection.

In conclusion, Configuration 3, featuring a thicker middle layer, stands out as the optimal layup, exhibiting the lowest deflection and the best overall performance compared to the other configurations by 230% to 90%. This underscores the critical role of layup design in enhancing the blast resistance of multi-layer LG panels.

### Effect on energy absorption

**Fig. 17:**
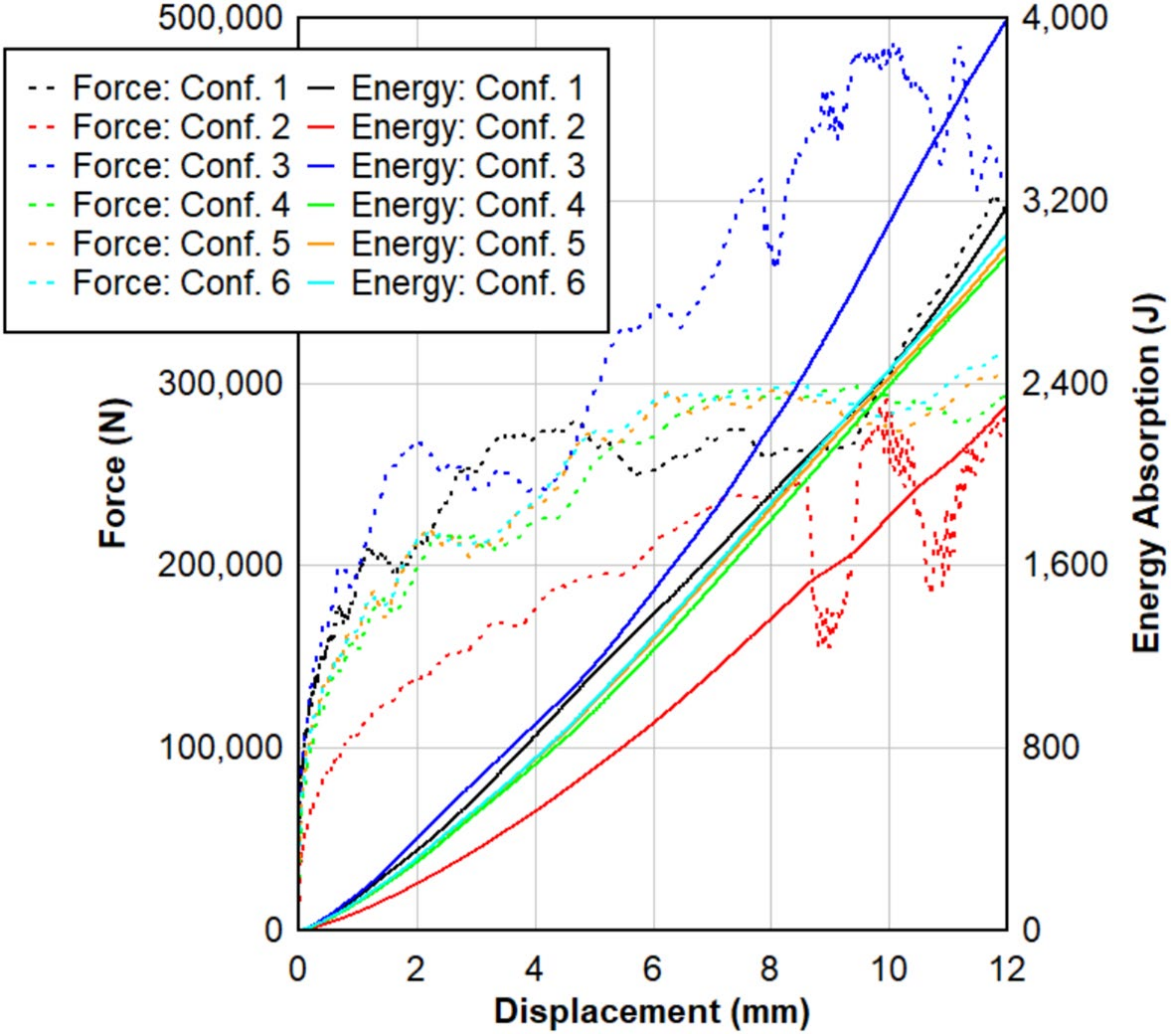
Force displacement dynamic response for different configurations including the energy absorption for each configuration.

Energy absorption is a key parameter in blast-resistant design, as it determines the panel’s ability to mitigate shock loads by dispersing impact energy^45^. The energy absorption behavior of six LG panel configurations, derived from finite element simulations, is summarized in Table 6 and illustrated in Fig. 17. Variations in interlayer and glass types significantly influence this performance metric. Systems with superior energy absorption reduce force transmission and structural deformation, offering enhanced protection against blast effects.

**Table 6:** Energy absorption results.

#	Configuration	Energy absorption (J)	Difference%
1	T_PVB_T	3173.6	–
2	T_EVA_T	2304.3	− 27.4
3	T_SG_T	3994.0	+ 25.8
4	T_TPU_T	2960.0	− 6.7
5	A_PVB_A	3004.0	− 5.3
6	H_PVB_H	3056.0	− 3.7

Based on Fig. 17, Configuration 3, which utilizes SG as the interlayer material between tempered glass plies, exhibited the highest energy absorption capacity at 3994.0 J, representing a 25.8% increase compared to Configuration 1 with PVB. Also, this can be shown in the force displacement response as SG sustains higher force levels over extended displacements. This enhanced performance is attributed to the stiffness and strong bonding characteristics of the SG interlayer, which effectively distributes stress and enhances resistance to deformation. In contrast, Configuration 2 showed the lowest energy absorption value (2304.3 J), with a 27.4% reduction compared to Configuration 1. The lower stiffness of the EVA interlayer contributes to increased deformability and less efficient energy dissipation under such loads. The energy absorption for Configuration 4 was slightly lower (2960.0 J), 6.7% less than Configuration 1, reflecting TPU’s moderate stiffness and deformability under dynamic loading.

Configurations 5 and 6 introduced variations in the glass type while keeping the PVB interlayer constant. As shown in Fig. 17, Configuration 5, which utilized annealed glass, absorbed 3004.0 J, a 5.3% reduction, whereas Configuration 6, which incorporated heat-strengthened glass, achieved 3056.0 J, only 3.7% below Configuration1. In summary, interlayer material selection plays a critical role in defining the energy absorption characteristics of LG panels, with SG showing superior blast mitigation. Additionally, glass type influences performance, with heat-strengthened glass slightly outperforming annealed glass when paired with the same interlayer.

## Conclusions

In this study, a comprehensive investigation into the blast performance of LG panels was conducted through numerical modeling and experimental validation. The research examined various factors, including interlayer type, hybridization, glass type, layer configuration, and layup design, to optimize the blast resistance of LG panels. The following conclusions were gained from this study:

- The validation of the FE model against Osnes et al.'s experimental data demonstrated a high degree of accuracy, with numerical predictions and glass fracture patterns closely matching experimental outcomes. Specifically, the maximum deflections of glass panels showed an average difference of less than 5% between numerical and experimental results across various blast pressures, providing strong validation for our numerical simulations.
- SG interlayers consistently outperform other types, exhibiting deflections 15% to 40% lower than PVB, EVA, and TPU counterparts across various blast pressures.
- Hybrid interlayers, such as T_EVA_PVB_EVA_T and T_EVA_SG_EVA_T, consistently underperform compared to pure PVB interlayers, exhibiting deflections 10% to 25% higher under similar blast pressures.
- Tempered glass panels experience deflections approximately 80% lower than annealed glass and 34% lower than heat-strengthened glass under similar blast pressures.
- Tailored configurations with thicker middle layers exhibit average decreases in deflection of around 50% compared to uniform thickness configurations.
- The SG interlayer system exhibited the highest energy absorption, outperforming Configuration 1 by 25.8%, highlighting its superior capacity for blast energy dissipation.

## Data Availability

All data generated or analyzed during this study are included in this published article.
